# A III-V-on-Si ultra-dense comb laser

**DOI:** 10.1038/lsa.2016.260

**Published:** 2017-05-19

**Authors:** Zhechao Wang, Kasper Van Gasse, Valentina Moskalenko, Sylwester Latkowski, Erwin Bente, Bart Kuyken, Gunther Roelkens

**Affiliations:** 1 Department of Information Technology (INTEC), Photonics Research Group, Ghent University-IMEC, Gent 9052, Belgium; 2 Center for Nano- and Biophotonics, Ghent University, Gent 9052, Belgium; 3 Electrical Engineering Department, Eindhoven University of Technology, Eindhoven 5600, Netherlands

**Keywords:** mode-locked lasers, near-infrared spectroscopy, optical frequency comb, semiconductor lasers, silicon photonics

## Abstract

Optical frequency combs emerge as a promising technology that enables highly sensitive, near-real-time spectroscopy with a high resolution. The currently available comb generators are mostly based on bulky and high-cost femtosecond lasers for dense comb generation (line spacing in the range of 100 MHz to 1 GHz). However, their integrated and low-cost counterparts, which are integrated semiconductor mode-locked lasers, are limited by their large comb spacing, small number of lines and broad optical linewidth. In this study, we report a demonstration of a III-V-on-Si comb laser that can function as a compact, low-cost frequency comb generator after frequency stabilization. The use of low-loss passive silicon waveguides enables the integration of a long laser cavity, which enables the laser to be locked in the passive mode at a record-low 1 GHz repetition rate. The 12-nm 10-dB output optical spectrum and the notably small optical mode spacing results in a dense optical comb that consists of over 1400 equally spaced optical lines. The sub-kHz 10-dB radio frequency linewidth and the narrow longitudinal mode linewidth (<400 kHz) indicate notably stable mode-locking. Such integrated dense comb lasers are very promising, for example, for high-resolution and real-time spectroscopy applications.

## Introduction

Optical frequency combs provide the long sought link between the radio frequency domain and the optical domain. They enable precise measurement of optical frequencies by the down-conversion to the measurable radio frequency (RF) domain. This technology has revolutionized the research field of frequency metrology and enabled the construction of optical clocks^1^. Recently, frequency combs have also been proven interesting to the field of spectroscopy^2^. Using the so-called dual-comb spectroscopic technique^3^, broadband absorption spectra can be measured with superior resolution and acquisition time compared with other techniques such as standard Fourier transform spectroscopy (FTIR). In this technique, one of the combs is used as an array of local oscillators to down-convert the lines of the probe comb to the RF domain. Because the resolution of the technique is inherently determined by the spacing of the lines of the combs, dense frequency comb generators are in high demand. Traditional means of comb generation rely on mode-locked fiber lasers^4^ and mode-locked titanium-sapphire lasers^5^. These bulky and costly lasers combine a broad comb bandwidth with a small line spacing of approximately 100 MHz. Reducing the size, cost and power consumption of such optical comb generators is of paramount importance to extend the application range of optical frequency combs. Therefore, a chip-scale, low-power consumption and low-cost comb source that can be electrically pumped and generates a broad comb with a small line spacing is desired.

Several approaches can be followed to reach this goal. There has been impressive progress in the field of quantum cascade laser frequency combs that operate in the mid-infrared and THz wavelength regions^6, 7, 8, 9^. In addition, the Intensity modulation of a CW laser source can generate frequency-agile combs^10^, although the frequency span is limited. Thus, a nonlinear spectral broadening step in fiber is mostly required. Recently, many studies focused on the integration of the so-called Kerr combs on a chip, which consist of a dispersion-engineered micro-resonator that is optically pumped by a strong continuous wave laser. The strong power build-up in the cavity enables strong nonlinear interactions, which generate a comb. Here, the spacing of the comb is determined by the free spectral range of the micro-resonator. Encouraging progress has been made in several material systems such as silicon nitride^11^, hydex glass^12^, silicon^13, 14^, and III-V AlGaAs resonators^15^. However, coupling the pump laser to the high Q-factor resonator can be difficult in real-life scenarios. The high-power external pump laser is also difficult to integrate on a chip, which makes the comb generator less compact and rugged. Finally, the typical large spacing of the comb lines (⩾10 GHz) is not attractive for high-resolution dual-comb spectroscopy. Although the comb line spacing can be reduced to below 10 GHz by extending the cavity length, one would require a much higher pump power to trigger the nonlinear process in the large cavity^16^.

Being the integrated counterpart of the fiber and solid-state mode-locked lasers (MLLs), monolithically integrated semiconductor MLLs have seen remarkable progress in the past decade^17, 18, 19, 20, 21, 22, 23, 24^. Mostly driven by telecom applications, semiconductor MLLs that operate at a repetition rate of 10–100 GHz are widely available. As previously discussed, spectroscopic applications require a smaller comb line spacing in the range of 100 MHz to 1 GHz. Particularly for gasses, the linewidth of an absorption line is in the GHz range. Mainly because of the high waveguide loss of III-V waveguides, which limits the cavity length, the comb line spacing (determined by the round-trip time in the cavity) of the integrated III-V mode-locked lasers remains at several GHz. Although this problem can be addressed by using an external cavity geometry^25^, the resulting device is not fully integrated.

In this letter, by leveraging the low optical loss of silicon waveguides, we present a III-V-on-silicon MLL that passively mode-locks at a record-low repetition rate of 1 GHz. The wide optical bandwidth (12-nm 10-dB bandwidth) and low repetition rate (1 GHz) result in an optical comb with over 1400 equally spaced narrow linewidth (<400 kHz) lines.

## Materials and methods

**Figure 1:**
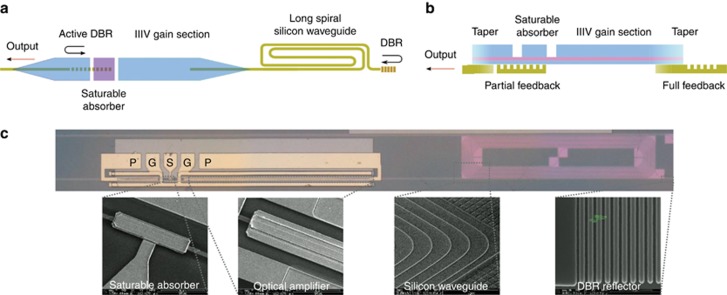
(**a**) Top and (**b**) side views of the anti-colliding MLL design. (**c**) Microscope image of the III-V-on-Si MLL. Insets: Scanning electron microscope images of various constituent parts of the laser.

The schematic design of the MLL is shown in Figure 1a. It consists of a long silicon spiral waveguide, two optical amplifiers (one of which acts as an amplifying spot-size converter to couple the light from the laser into an external silicon waveguide circuit) that are separated by a saturable absorber, and two distributed Bragg reflectors (DBR), which form the mirrors of the cavity. As shown in Figure 1b, by implementing the saturable absorber (SA) above the output DBR reflector, the MLL works in an anti-colliding mode, which promises higher output power, lower timing jitter, and better RF spectral purity^26, 27^ than a colliding-pulse MLL. Both optical amplifiers and saturable absorber are realized by heterogeneously integrating an InGaAsP-based multi-quantum well (MQW) epitaxial stack on top of a 400-nm silicon waveguide layer. The details of the epitaxial layer stack can be found in reference 28. A more detailed description of the heterogeneous integration process based on adhesive die-to-wafer bonding can be found in reference 29. The total length of the optical amplifier is 800 μm, and a 40-μm-long SA is isolated from the amplifier by two 15-μm wide electrically isolating slots in the p contact layer. Because of the low loss of the passive SOI waveguide (~0.7 dB cm^−1^), we can implement a long passive cavity with a length of 37.4 mm, which permits the 1 GHz repetition rate.

## Results and discussion

**Figure 2:**
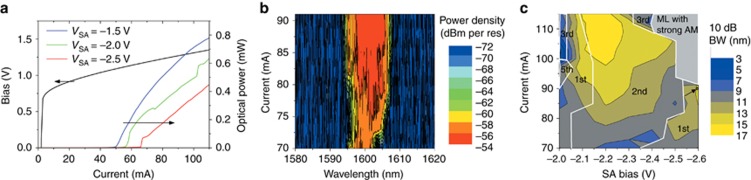
(**a**) Measured Current-Voltage (IV) curve and Light-Current (LI) curves for different SA bias. (**b**) Measured optical spectra as a function of the injected current in the gain sections, when the SA is biased at −2.6 V. (**c**) Mapping of 10-dB optical bandwidth as a function of the SOA injection current and SA bias. Different harmonic mode-locking regions are marked. The black dot indicates the optimal operation point for the 1-GHz laser operation. AM, amplitude modulation; ML, mode-locking.

The laser characterization results were obtained with the sample on a thermoelectric cooler, which maintained the laser substrate at 20 °C. The coupling to single-mode optical fiber was realized using a fiber-to-chip grating coupler with a coupling loss of 10 dB. The measured IV and LI curves at different SA bias are plotted in Figure 2a. The kinks on the LI curves are attributed to the parasitic reflections from the grating coupler. Because of the low-loss silicon waveguide, a relatively low threshold current (60 mA) was achieved even when the SA bias was reversely biased. The passive mode-locking operation occurs at an SA bias lower than −2 V. As an example, Figure 2b shows the evolution of the optical spectrum as a function of the injection current of the optical amplifier. Here, the SA was biased at −2.6 V. At this reverse bias, the laser output spectrum was broad even when the current injection was immediately above the threshold. When the current increased, the 3-dB bandwidth of the spectrum significantly broadened and reached a maximum of 10 nm. Figure 2c maps the optical spectra as a function of the injection current and SA bias. Because the optical spectrum is not always notably flat, it is more practical to measure the 10-dB optical bandwidth^20, 30^.

As observed, over a large operation window, the MLL generates a broad optical spectrum of more than 10 nm wide with a maximum bandwidth of 15.8 nm. In the case of the quantum-well semiconductor MLL, the comb span is mainly determined by the cavity dispersion and gain competition among different optical modes. To broaden the comb span, one may use quantum-dot (QD) or quantum-dash materials, which provide a broader gain spectrum and suffer less from gain competition because of the inhomogeneous broadening. For quantum-well materials, by incorporating an intra-cavity filter to equalize the threshold gain of different longitudinal modes, it is possible to reduce the gain competition between optical modes, and a much wider comb span can be expected^31, 32^. Nonlinear processes in the laser cavity, such as self-phase modulation, can also be used to extend the bandwidth^33^. The waveguide dispersion of the current MLL design was not compensated (see more details of the dispersion characterization below). Further optimization of the cavity dispersion can further broaden the comb span, which is particularly important for spectroscopy applications^34, 35^.

In Figure 2c, different operation regions for different harmonic mode-locking orders are also marked. In a large operation window, the laser is mostly mode-locked at the 2nd harmonic order, that is, with a repetition rate of 2 GHz. The laser can even operate at higher harmonic orders (3rd and 5th) when the injecting current is high and the reverse bias is relatively low (Figure 2c). When both injection current and reverse bias are large, the lasing spectra significantly shifts to shorter wavelengths, and notably strong amplitude modulations (AM) are found to considerably degrade the mode-locking stability. The favored fundamental mode-locking occurs only when the injecting current is low and the SA reverse bias is high (see the right-bottom corner of Figure 2c). Although the laser can be mode-locked at its fundamental repetition rate when the SA is biased at approximately −2 V, modulation on top of the RF spectra occurs because of the relaxation oscillation of the laser. Therefore, in the following discussion, we will focus on the optimal operation point as indicated by the black dot in Figure 2c.

**Figure 3:**
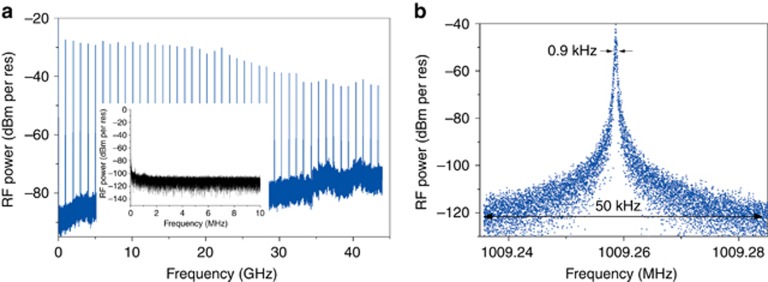
(**a**) RF spectrum of the generated pulse train (RBW 300 KHz, VBW 10 KHz) when the laser is operated at the optimal operation point of Figure 2c. Inset: enlarged RF spectrum in the frequency range of 1–10 MHz (**b**) Detail of the 1-GHz RF tone (RBW 10 Hz, VBW 10 kHz).

Figure 3a shows the RF spectrum of the generated pulse train at the optimal operation point (*I*_current_=91 mA, *V*_SA_=−2.6 V, indicated by the dot in Figure 2c). The strong fundamental tone that is 55 dB above any spurious peaks or noise floor and the clean RF spectrum in the low-frequency range (see the inset) indicate that there is notably little residual amplitude modulation of the pulse train. A more detailed plot of the fundamental tone is shown in Figure 3b. It was recorded with a resolution bandwidth and a video bandwidth of 10 kHz. The exact repetition rate of the MLL is 1009.259 MHz. The 10-dB linewidth measured from the spectrum is below 900 Hz. The laser presents similar noise properties when it is locked at higher-order harmonics, and the measured RF linewidth is in the range of a few kHz. As required for optical comb spectroscopy, to stabilize the repetition rate of the MLL, one can realize the hybrid mode-locking of the laser by supplying an extra RF input signal to the SA, which will be discussed later.

**Figure 4:**
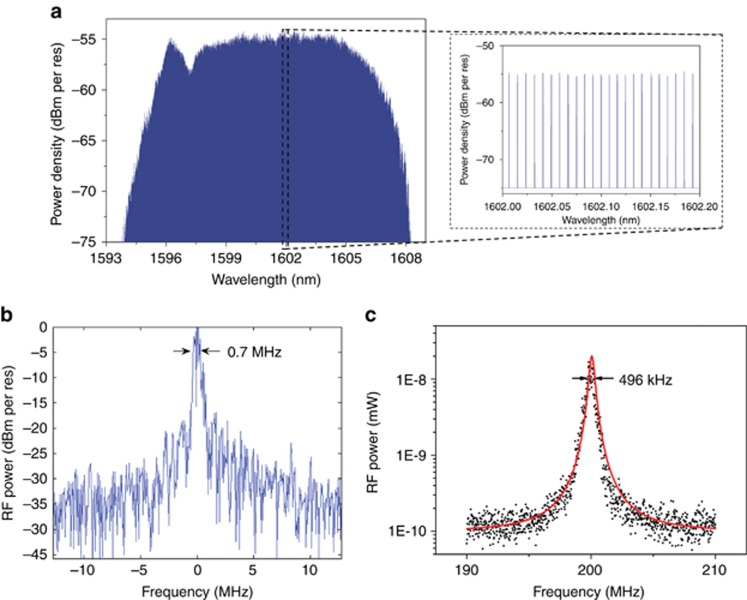
(**a**) An optical comb generated by the passively locked 1 GHz MLL. Inset: a detail of evenly spaced optical modes in the comb. (**b**) Beat note between the optical comb and the tunable laser at a wavelength of 1600 nm. (**c**) Measured optical linewidth of the MLL using the delayed self-heterodyne method, which indicates an optical linewidth below 250 kHz. The black dots are the measured data, and the red curve is the corresponding Lorentzian fitting.

Figure 4a shows the optical comb spectrum measured by a high-resolution optical spectrum analyzer (5 MHz resolution). The MLL is set at the identical optimal operation point as indicated in Figure 2c. A more detailed image of the evenly spaced optical modes is shown in the inset. The large optical bandwidth (>10 nm) and the small repetition rate (1 GHz) result in an optical comb with more than 1400 optical lines. The small ripples on top of the optical comb are attributed to the fact that the residual reflections from the fiber-to-chip grating coupler and the III-V-to-silicon taper form a Fabry-Perot cavity, which introduces wavelength-dependent transmission. The origin of the relatively strong peak on the blue side of the comb is under investigation, although it can be attributed to the self-phase modulation process considering the high optical intensity in the highly nonlinear sub-micron silicon waveguides.

The linewidth of the longitudinal modes of the laser was first measured by heterodyning the output of the MLL with a CW OPO (Argus 2400 sf, 50 kHz linewidth) on a 26-GHz bandwidth photodiode with TIA and analyzing the output with an electrical spectrum analyzer (Keysight EXA N9010). The temperature of the TEC slowly fluctuated as follows: a temperature change of 0.1 degree Celsius corresponded to an optical frequency shift of 1.5 GHz at 1600 nm. Thus, the generated heterodyne beat note slowly drifted, which substantially broadened the measured linewidth as shown in Figure 4b. The signal-to-noise ratio is poor mainly because of the low-resolution and fast-scan-speed setting of the spectrum analyzer to capture an instantaneous image of the drifting beat note. To cancel the effect of such temperature fluctuation, a delayed self-heterodyne method was used. A narrow spectral band of the MLL output (0.5 nm wide optical spectrum centered at 1600 nm) was split into two arms. In one arm, the optical signal went through a 5-km-long single-mode fiber, whereas a 200-MHz frequency shift was introduced in the other arm. The beat note measured by mixing the two optical signals is plotted in Figure 4c. A fairly narrow beat note with below-500-kHz 3-dB bandwidth was obtained, which indicates an optical linewidth below 250 kHz. Note that it is not possible to filter out a single longitudinal mode for the linewidth measurement. Therefore, the current beat note was obtained by beating ~60 optical modes (in a 0.5-nm bandwidth). Therefore, the intrinsic optical linewidth of each longitudinal mode may be even smaller. The identical measurement was performed by scanning the central wavelength of the filter from 1598 to 1608 nm, and an optical linewidth below 400 kHz was obtained across the entire wavelength range. The optical linewidth could be further reduced by implementing an optical phase-locked loop to lock the MLL to an external narrow linewidth laser, which could be potentially co-integrated with the MLL^36^.

**Figure 5:**
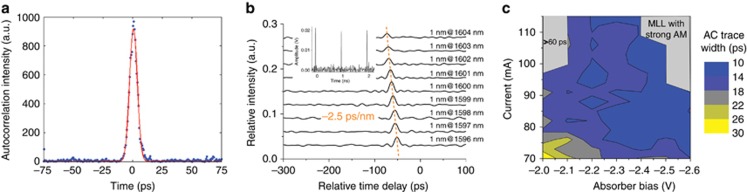
(**a**) An auto-correlation trace and the corresponding fit of the MLL output. (**b**) The recorded pulse traces by a real-time oscilloscope for different parts of the optical comb. The inset shows a time trace of the pulse trains, which were recorded using a real-time oscilloscope. (**c**) Mapping of the auto-correlation trace width over the current injection and SA bias. The arrow indicates the optimal operation point.

From the measured auto-correlation (AC) trace of the pulse train (Figure 5a, the laser operates at the identical optimal point of Figure 2c), the fitted pulse width was ~7 ps, which indicates that the generated pulse was not transform limited. Similar to the technique in reference 37, using a tunable filter and an EDFA, we amplified and fed different slices of the optical comb (with a 1-nm bandwidth) into a high-speed photodiode and recorded the real-time pulse traces using a 160-Giga samples per second real-time oscilloscope. A typical time trace of the pulse train is shown in the inset of Figure 5b. An overall chirp of −2.5 ps nm^−1^ was derived, which also included the chirp introduced by the EDFA (−1 ps nm^−1^, which was measured by using a standard time-of-flight dispersion measurement). The chirp management of the laser cavity design or an external dispersion-compensating optical fiber can further reduce the pulse width, but it is not an important characteristic of an optical comb for spectroscopy. To fully describe the pulse width evolution, we plotted the AC trace width as a function of the injection current and SA bias in Figure 5c. The AC trace width was maintained below 15 ps over a large operation window, whereas the pulse width was considerably broader (>60 ps) for the operation region at the top-left corner of the figure, which corresponds to the small optical bandwidths in Figure 2c.

**Figure 6:**
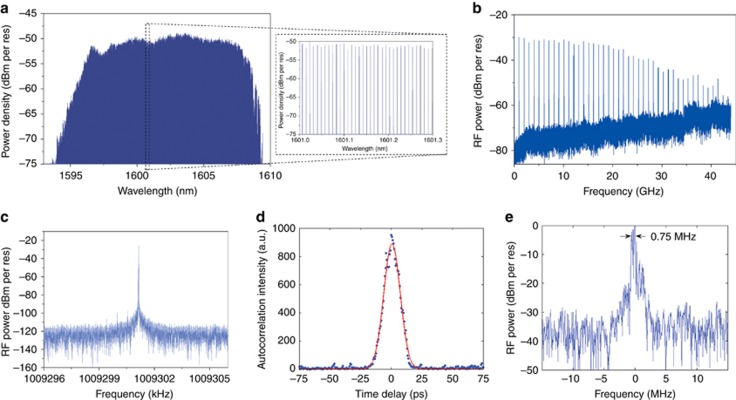
(**a**) High-resolution optical spectrum of the optical comb when the MLL is hybrid mode-locked (inset: magnification of the optical spectrum). (**b**) RF spectrum of the generated pulse train. (**c**) Fundamental RF peak over a span of 10 kHz. (**d**) An auto-correlation trace of the output pulse when the laser is hybrid mode-locked. (**e**) Beat note between the optical comb and the tunable laser at a wavelength of 1607 nm, which indicates that the linewidth of the optical mode is smaller than 1 MHz.

Two degrees of freedoms, that is, the repetition rate and the offset frequency, of the presented mode-locked laser must be stabilized to realize a frequency comb. The hybrid mode-locking of the laser was performed by supplying an RF signal to the SA to stabilize the repetition rate. The corresponding optical comb is shown in Figure 6a. The MLL operation conditions were 91 mA current injection, −2.6 V SA bias, 1.00930116 GHz RF input frequency and 8 dBm RF input power. Compared with the optical comb generated by the passive mode-locking (Figure 4a), the peak at the blue side of the spectrum was less pronounced when the MLL was hybrid mode-locked (Figure 6a). More importantly, the optical comb slightly extended to the red side instead of collapsing into a much narrower comb when the laser was hybrid mode-locked^19^. An analysis of the pulse train in the RF domain (Figure 6b) reveals that the FWHM of the fundamental RF tone was sub-Hz, which proves that the line spacing of the optical comb can indeed be well stabilized (see the RF peak in Figure 6c). The AC trace of the output pulse is shown in Figure 6d, which shows a slightly broadened pulse width compared with the passive mode-locking case. In addition, the measured linewidth of the individual comb lines was again less than 1 MHz across the entire comb (Figure 6e), which is comparable to the measured linewidths when the laser was passively mode-locked (Figure 4). The measurement results prove that it is indeed possible to stabilize an optical comb without reducing the overall optical bandwidth, which is notably promising for high-resolution, high-speed spectroscopic applications.

In addition to the repetition rate, the other degree of freedom of the laser, which is the offset frequency *f*_ceo_^24^, must be stabilized. The widely explored self-referencing-based stabilization approach is not feasible here because of the lack of an octave-spanning spectrum without further nonlinear spectral broadening^14, 38^. Furthermore, f_ceo_ can also be stabilized by the electronic feedback modulation of the injected current in the laser using an external reference^39^ or by external laser injection^40^, as we recently demonstrated. The electronic feedback scheme is more elegant because it is more robust than optical injection locking and does not require optical isolation. Our heterodyne measurements (beating the MLL output with the narrow linewidth emission from a CW OPO) show that the fine tuning of the laser injection current modifies the offset frequency. The next step of the work is the implementation of an integrated electronic feedback loop to control *f*_ceo_ by modulating the injection current.

It is worth noting that in some cases, it is not critical to stabilize the absolute offset frequencies of the two combs for dual-comb spectroscopy. Using an external reference, one can track the drift of the two frequency combs and implement signal processing to compensate for the instabilities of free-running lasers^41^.

The performance of the presented mode-locked laser is compared with other demonstrations in the literature in Table 1. For the first time, the demonstrated MLL combines all favored properties in terms of a wide optical spectrum, a low repetition rate, a narrow optical linewidth and a narrow RF linewidth, which can be further stabilized without narrowing the optical spectrum. All of these properties successfully demonstrate an integrated dense comb laser, which is notably promising for high-performance, compact and low-cost spectroscopic sensing applications.

Although the current communication wavelength range is less interesting for sensing applications, the hybrid MLL configuration extends the wavelength range to, for example, the shortwave infrared by heterogeneously integrating other gain materials to silicon. For example, we recently demonstrated a III-V-on-silicon 2.3 μm laser based on type-II quantum-well materials^42^. The versatility of the integration scheme enables the exploration of new wavelength regions while enjoying the benefits of low-loss silicon waveguides. One can also co-integrate a highly nonlinear, dispersion-engineered waveguide (for example, AlGaAs^15^, Si^13, 14^, SiN^11^) for further comb broadening. Thus, one can expect to address wavelength regions beyond the limitations imposed by the optical gain material.

## Conclusions

We have successfully demonstrated an integrated III-V-on-silicon mode-locked laser that passively mode-locks at a record-low repetition rate of 1 GHz. Because of the low loss of the passive silicon waveguide, the sub-kHz 10-dB linewidth of the fundamental RF tone indicates low phase noise. The over 10-nm wide optical comb with a line spacing of only 1 GHz consists of more than 1400 densely and evenly spaced optical lines with below 400-KHz optical linewidth. Hybrid mode-locking stabilizes the repetition rate of the optical comb without negatively affecting the bandwidth and linewidth of the individual comb lines. The fully integrated comb laser provides unique advantages of compactness, robustness, low power consumption and low cost, which enable cost-sensitive applications such as mobile spectroscopic analysis.

## Figures and Tables

**Table 1:** Comparison between the presented mode-locked laser and the state-of-the-art results in the literature

	*Mode-locking mode*	*Repetition rate*	*10 dB optical bandwidth*	*3 dB optical linewidth*	*10 dB RF linewidth*	*AC trace width*	*# Comb lines within 10 dB bandwidth*
[17]	Hybrid	1.0385 GHz	<1 nm	70 MHz	500 kHz	36 ps	<110
[18]	Passive	2.1 GHz	1 nm	—	>1.3 MHz	15.4 ps	120
[19]	Active	0.927 GHz	0.1 nm	—	—	200 ps	12
	Passive	1.99 GHz	1 nm		>14 kHz	40 ps	126
[20]	Passive	10.16 GHz	8.7 nm	—	>15 kHz	32.8 ps	110
[21]	Passive	20 GHz	15 nm	900 MHz	2.4 MHz	3.8 ps	90
[22]	Passive	2.5 GHz	4 nm	—	18.9 kHz	15 ps	210
[23]	Passive	16.6 GHz	—	1 GHz	<2 kHz	—	—
This work	Passive	1.009 GHz	12 nm	<400 kHz	0.9 kHz	15 ps	1400
	Hybrid	1.009 GHz	13 nm	<400 kHz	<1 Hz	15 ps	1500
